# Cardiac Rehabilitation in the Modern Era: Evidence, Equity, and Evolving Delivery Models Across the Cardiovascular Spectrum

**DOI:** 10.3390/jcm14155573

**Published:** 2025-08-07

**Authors:** Anna S. Mueller, Samuel M. Kim

**Affiliations:** 1 Department of Medicine, Mount Sinai Morningside/West, Icahn School of Medicine at Mount Sinai, New York, NY 10025, USA; 2 Department of Cardiology, NewYork-Presbyterian Hospital/Weill Cornell Medicine, New York, NY 10065, USA; smk9018@med.cornell.edu

**Keywords:** CR, secondary prevention, health disparities, innovative care models, healthcare policy

## Abstract

CR is a cornerstone of secondary prevention for cardiovascular disease, offering well-established benefits across mortality, hospital readmission, functional capacity, and quality of life. Despite Class I guideline endorsements and decades of supporting evidence, CR remains vastly underutilized, particularly among women, racial and ethnic minorities, older adults, and individuals in low-resource settings. This review synthesizes the current evidence base for CR, with emphasis on disease-specific benefits across different cardiovascular diseases, and highlights recent data on its role in expanding populations, including patients with HFpEF, older adults, patients with advanced heart failure, and those undergoing transcatheter interventions. We also examine persistent barriers to CR access and participation, including system-level and referral limitations, as well as patient-level disparities by age, sex, race and ethnicity, and socioeconomic status. Building on this, we explore innovative delivery models and recent policy initiatives such as hybrid programs and reimbursement reform, all designed to expand access, promote equity, and modernize CR delivery. The findings underscore the need for continued investment, advocacy, and innovation to ensure equitable access to CR and its life-saving benefits across the full cardiovascular care continuum.

## 1. Introduction

Cardiovascular disease remains the leading cause of death in the United States, with over 18 million adults currently living with some form of heart disease [1]. As advances in medical and interventional therapies continue to prolong survival, a growing number of individuals are living with chronic cardiovascular conditions. As a result, the focus of care has shifted beyond just extending lifespan toward improving quality of life, functional capacity, and long-term well-being.

Cardiac Rehabilitation (CR) is a comprehensive, guideline-recommended, multidisciplinary program that includes supervised exercise training, risk factor modification, up-titration of medical therapy, management of comorbidities, psychosocial support, and lifestyle counseling. It is consistently identified as one of the most effective interventions for secondary prevention, with benefits that extend across clinical outcomes, patient-reported quality of life, and healthcare utilization. Participation in CR programs has been associated with a range of benefits including reduced hospital readmissions, improved exercise capacity, decreased depressive symptoms, and enhanced return to work and daily activities, with the strength of supporting evidence varying across different cardiac populations [2]. Despite this strong evidence base and Class I guideline recommendations, CR remains severely underutilized [3]. National data show participation rates averaging just 19.6%, ranging from 5.7% to 42% by state [4]. Utilization is even lower among women, racial and ethnic minorities, older adults, individuals with caregiving responsibilities, those with multiple comorbidities, and patients facing barriers such as limited insurance coverage or geographic access to CR programs [3,5].

This persistent gap highlights a critical opportunity: to rethink delivery models, increase awareness among providers and patients, and expand access through innovative strategies such as virtual or hybrid CR programs. As more patients live longer with heart disease, CR is not solely about extending survival, but about improving functional status, emotional well-being, and overall quality of life.

## 2. Guideline Recommendations and Components of CR

CR is strongly endorsed by major professional societies, including the American Heart Association, American College of Cardiology, and the European Society of Cardiology [6,7,8] for a wide range of cardiovascular conditions, underscoring its role as a critical component of secondary prevention. Accordingly, Medicare Part B currently covers CR programs for beneficiaries with any of the following qualifying conditions:

1. Acute myocardial infarction within the past 12 months;
2. Percutaneous coronary intervention or coronary artery bypass grafting;
3. Stable angina pectoris;
4. Heart valve repair or replacement;
5. Heart transplantation;
6. Chronic stable heart failure with reduced ejection fraction (NYHA Class II–III, LVEF ≤ 35%).

The most recent joint update from the American Heart Association (AHA) and the American Association of Cardiovascular and Pulmonary Rehabilitation (AACVPR) reaffirms that CR is far more than just supervised exercise. It is a structured, multidisciplinary program that addresses the full spectrum of physical, psychological, and behavioral needs [9]. Core components include

1. Supervised aerobic and resistance training, ideally guided by cardiopulmonary exercise testing (CPET), which allows for individualized exercise prescription. The FITT-VP principle (Frequency, Intensity, Time, Type, Volume, and Progression) is often used to structure training recommendations [10].
2. Cardiovascular risk factor management, including blood pressure, cholesterol (with attention to achieving LDL-C targets), diabetes, and smoking cessation.
3. Optimization of medical therapy, including the up-titration of guideline-directed drugs in conditions like heart failure.
4. Nutritional counseling and weight management.
5. Psychosocial support, including depression screening and stress management.
6. Lifestyle counseling to promote physical activity, healthy habits, and medication adherence.
7. Assessment for Cardiac Implantable Electronic Devices (CIEDs) in eligible patients or those who may benefit from device therapy.
8. Ongoing program evaluation and quality assurance metrics to ensure effectiveness and safety.

These components are designed to be personalized to the patient’s clinical condition, goals, and barriers to care. CR is delivered by a multidisciplinary team, typically led by a cardiologist and including nurses, exercise physiologists, dietitians, behavioral health specialists, and, in some cases, pharmacists and social workers. Programs are generally structured around 36 sessions over 12 weeks, though duration and intensity can be adapted to patient needs. CR can be delivered in center-based, home-based, virtual, or hybrid models, allowing flexibility in program access. In contrast to the predominantly outpatient, hospital-based CR model used in the U.S., many European countries offer inpatient CR through specialized rehabilitation hospitals, particularly in Western and Northern Europe [11,12,13,14,15]. Regardless of format, adherence to the core components is essential to ensure clinical benefit.

## 3. Mechanism of Benefit

Exercise training (ET), the cornerstone of CR, exerts broad physiologic effects that collectively enhance cardiovascular function and exercise capacity.

Cardiac and Hemodynamic Effects: ET reduces resting and submaximal heart rates, lowers exercise blood pressure, and improves myocardial electrical stability [16,17]. Though improvements in left ventricular ejection fraction are modest, functional gains occur through enhanced cardiac output, better ventricular filling, and favorable remodeling [18]. In heart transplant recipients, ET restores sympathetic innervation and improves chronotropic competence [19].

Neurohormonal and Autonomic Modulation: ET attenuates sympathetic activation, decreases circulating N-terminal pro–B-type natriuretic peptide, and enhances vagal tone [16]. These adaptations mirror beta-blocker therapy, improving ischemic thresholds and reducing arrhythmogenic risk by modulating beta-receptor sensitivity and norepinephrine levels [20].

Peripheral and Skeletal Muscle Adaptations: Aerobic exercise improves endothelial function, enhances skeletal muscle oxidative capacity, and increases mitochondrial biogenesis [21,22]. These changes support greater oxygen extraction, delay anaerobic metabolism, and improve ventilatory efficiency, resulting in higher VO_2_ peak and longer exercise duration [22,23].

Vascular and Anti-Atherosclerotic Effects: ET promotes endothelial regeneration, vasodilation, and angiogenesis through the upregulation of factors such as HIF1α and VEGF [24]. These adaptations enhance coronary flow reserve, improve microvascular perfusion, and offer protection against ischemia [20]. In transplant recipients, ET has also been shown to reduce inflammation and oxidative stress, delaying the progression of cardiac allograft vasculopathy [25].

Metabolic and Inflammatory Effects: Exercise promotes weight loss, preserves lean body mass, and reduces visceral and hepatic fat through increased energy expenditure and enhanced fat oxidation, even in the absence of significant changes in total body weight [26]. Regular physical activity also improves insulin sensitivity and lipid profiles while reducing systemic inflammation [27,28]. In addition, CR programs support smoking cessation through integrated behavioral counseling and pharmacologic interventions, further contributing to overall cardiovascular risk reduction [29].

## 4. Clinical Evidence by Disease Type

### 4.1. Coronary Artery Disease (CAD)

CR significantly enhances recovery in patients with CAD across the spectrum of care. A Cochrane review of 63 RCTs showed that, following myocardial infarction (MI), CR participants improved exercise duration by 1.5 min and peak VO_2_ by 1.8 mL/kg/min compared to usual care [2]. Among patients post-coronary artery bypass grafting (CABG), CR led to a 58 m increase in 6 min walk distance and a +1.6 MET improvement in exercise capacity [30]. Following PCI, CR has been associated with gains in exercise duration, metabolic profile, and functional capacity across age groups [31]. Notably, in stable CAD, aerobic training improved maximal workload by 36% and delayed the ischemic threshold more effectively than PCI alone, underscoring the therapeutic potency of exercise [32]. Moreover, quality-of-life benefits are well-established. A 2021 Cochrane review of 85 RCTs (23,430 patients) found small but consistent improvements across multiple SF-36 domains, including physical functioning, vitality, and mental health, in CR participants versus controls [33]. The UPBEAT trial demonstrated comparable effects of exercise and SSRIs on depressive symptoms in CAD patients [34]. Beyond psychological health, CR facilitates reintegration into daily life. A Dutch national cohort study of 24,509 patients showed higher odds of return to work among CR participants, rising progressively from 6 to 12 months post-event [35]. CR also improves long-term clinical outcomes. Early studies and meta-analyses have shown 13–42% reductions in all-cause mortality, depending on follow-up duration and population studied [36,37]. CR has also been shown to reduce both all-cause and cardiac-specific hospitalizations, with a number needed to treat of 12 to prevent one hospital admission [33,38]. A Medicare analysis of over 600,000 patients demonstrated significantly lower 5-year mortality among CR participants, with a clear dose–response relationship—those attending ≥25 sessions had the greatest benefit [39,40].

### 4.2. Heart Failure with Reduced Ejection Fraction (HFrEF)

CR confers substantial benefits in patients with HFrEF, including improvements in functional capacity, quality of life, and clinical outcomes. Early trials reported increases of up to 15–17% in peak VO_2_ and significant gains in 6MWD, though these were limited by small sample sizes, single-center designs, and lack of GDMT [41]. The landmark HF-ACTION trial, the largest multicenter RCT in HFrEF patients on contemporary GDMT, demonstrated modest but statistically significant improvements in peak VO_2_ (+0.4 mL/kg/min) and exercise duration (+1.3 min) at 3 months, though changes in 6MWD at 12 months were not significant [42]. Health status also improved significantly in HF-ACTION, with gains across physical limitation, symptom burden, social functioning, and overall quality of life domains of the Kansas City Cardiomyopathy Questionnaire. These improvements were evident within three months and sustained long-term. Moreover, a post hoc analysis showed even greater benefits among participants with high adherence to the exercise regimen [42]. Similarly, the REHAB-HF study, which evaluated a tailored rehabilitation program in older adults hospitalized with heart failure, found that CR reduced anxiety, depression, and stress, while enhancing social connectedness likely due to the combined benefits of group interaction and personalized support [43]. In terms of clinical outcomes, both HF-ACTION and EJECTION-HF evaluated the effect of CR on major events. The EJECTION-HF trial found no statistically significant reduction in all-cause death or hospital readmission at 12 months with exercise training. However, exploratory subgroup analysis suggested a possible signal of benefit in patients under the age of 70. In addition, participants who adhered to the prescribed exercise regimen experienced significantly lower rates of death or readmission compared to those who did not (55.5% vs. 75.9%, *p* = 0.008) [44]. In the HF-ACTION trial, the unadjusted analysis showed no statistically significant difference in all-cause mortality or hospitalization (hazard ratio 0.93; *p* = 0.13). However, after prespecified adjustment for baseline variables such as left ventricular ejection fraction, exercise capacity, and depression, exercise training was associated with a significant reduction in all-cause mortality or hospitalization (hazard ratio 0.89; *p* = 0.03) [42]. Observational data further support these findings. A large Japanese cohort study *(n* = 3277) found that participation in multidisciplinary outpatient CR was associated with a 33% reduction in all-cause mortality (adjusted HR 0.67; 95% CI, 0.55–0.82; *p* < 0.001) and an 18% reduction in HF-related rehospitalization (adjusted HR 0.82; 95% CI, 0.70–0.97; *p* = 0.018) [45]. Collectively reinforcing these findings, a large retrospective analysis from a global federated health research network (n > 40,000), primarily conducted in the U.S., demonstrated that exercise-based CR was associated with significantly lower odds of all-cause mortality (OR 0.58; 95% CI, 0.54–0.62; *p* < 0.001), hospitalization (OR 0.74; 95% CI, 0.71–0.77; *p* < 0.001), stroke (OR 0.63; 95% CI, 0.51–0.79; *p* < 0.001), and atrial fibrillation (OR 0.47; 95% CI, 0.40–0.55; *p* < 0.001) among patients with HF [46].

A 2023 Cochrane review confirmed likely reductions in all-cause hospitalization (RR 0.69) and suggested a mortality benefit with long-term follow-up [47]. Nonetheless, some patients do not experience improvements in peak VO_2_ following CR and are considered “non-responders.” [48]. Evidence from prospective cohorts indicates that non-response is associated with more than a twofold increased risk of mortality or hospitalization in subsequent years [49]. Predictors of non-response include older age, higher comorbidity burden, lower baseline muscle strength, and inadequate exercise intensity [48,49,50]. Recognizing non-response may serve as a marker of disease severity and highlights the importance of early identification and tailored strategies to maximize benefit.

### 4.3. Valvular Heart Disease

Studies examining CR in patients with valvular heart disease remain relatively limited. Most available data come from retrospective cohorts or uncontrolled prospective studies that often combine multiple valve pathologies, which restricts disease-specific conclusions. Only three randomized controlled trials involving 295 patients were published between 1987 and 2016 [51], and all reported significant improvements in exercise capacity. In the CopenHeart VR trial, two-thirds of whom had undergone aortic valve replacement, structured exercise training increased peak VO_2_ from 22.5 to 24.8 mL/kg/min [52]. Similarly, the ENERGY trial, which evaluated a structured rehabilitation program in patients undergoing transcatheter aortic valve replacement (TAVR), demonstrated significant improvements in peak VO_2_ compared to usual care [53]. Although neither trial showed significant improvements in global quality of life, observational studies have reported more favorable results. A 2024 meta-analysis that included both randomized and observational studies found that CR was associated with meaningful gains in the physical component of quality of life and reductions in anxiety, although no consistent benefit was observed for depression or overall mental health scores [54]. While randomized trial data remain sparse, real-world evidence suggests substantial cardiovascular benefit. In a Medicare cohort of more than 41,000 patients undergoing valve surgery, CR participation was associated with a 61% relative reduction in one-year mortality (HR 0.39; 95% CI, 0.35–0.44; *p* < 0.001) and a 34% reduction in hospitalizations (HR 0.66; 95% CI, 0.63–0.69; *p* < 0.001) [55]. 

A Dutch national study of over 3.3 million insured individuals similarly reported a 45 percent lower mortality risk in patients who participated in CR after valve and/or bypass surgery, with consistent benefits across valve types and age groups [56]. Although a 2021 Cochrane review of 364 patients did not demonstrate a mortality benefit, likely due to small sample size, the growing body of real-world data supports CR as an important intervention to reduce cardiovascular mortality and readmissions in this expanding population [57].

### 4.4. Heart Transplant

Although traditionally underrepresented in CR research, accumulating evidence supports the role of structured exercise-based CR in heart transplant recipients. One of the earliest RCTs demonstrated significant improvements in peak VO_2_ (+3.1 mL/kg/min) and muscle strength (>30%) after 12 weeks of CR [26]. The HITTS trial later compared high-intensity interval training (HIIT) to moderate-intensity training, showing superior gains in peak VO_2_ at one year (+4.6 vs. +2.5 mL/kg/min), which were sustained at three years alongside preserved muscle strength [19]. While short-term improvements in anxiety were not observed, a five-year follow-up reported significantly fewer anxiety symptoms in the HIIT group, suggesting long-term psychosocial benefits [58]. A 2017 Cochrane review of three small trials found no significant short-term improvements in quality of life, citing low certainty of evidence and methodological limitations [59], underscoring the need for more robust studies. Regarding cardiovascular outcomes, observational studies suggest potential benefit, though randomized data remain limited. In a national Medicare cohort, CR participation was associated with a 29% lower 1-year readmission rate (adjusted HR 0.71; 95% CI: 0.58–0.87) [60]. Similarly, Uithoven et al. found that transplant recipients attending ≥23 CR sessions had a nearly 60% lower incidence of major adverse cardiac events including stroke, PCI, HF hospitalization, MI, acute rejection, or death compared to those with lower attendance, even after adjustment for clinical variables [61]. Overall, these results highlight CR as a safe, feasible, and potentially impactful intervention for transplant recipients, while reinforcing the need for high-quality trials to confirm its long-term benefits.

## 5. Emerging Evidence in Expanding Populations

### 5.1. Heart Failure with Preserved Ejection Fraction (HFpEF)

HFpEF accounts for approximately 50% of all heart failure cases and is particularly prevalent among older adults and women. Despite its high burden and rising prevalence, HFpEF remains excluded from current Centers for Medicare & Medicaid Services coverage for CR. This policy gap persists even as a growing body of evidence supports the benefits of exercise-based interventions in this population. Support for CR stems in part from earlier data in patients with HFrEF. In recent years, an increasing number of studies have focused on evaluating the role of exercise training in patients with HFpEF, a population for whom evidence is still emerging. One of the earliest exercise trials in this population evaluated aerobic exercise and caloric restriction in obese older adults with HFpEF. This single-center randomized trial showed that both interventions improved peak VO_2_ and quality of life, with the greatest benefits observed in participants who received both [62]. Building on these results, the OptimEx-Clin trial, the largest conducted to date, was a multicenter randomized controlled study that compared moderate-intensity continuous training (MCT) and HIIT in patients with HFpEF [63]. Both exercise modalities led to modest short-term improvements in VO_2_ peak. However, the differences were not statistically significant, and neither exercise arm met the study’s prespecified minimal clinically important difference of 2.5 mL/kg/min. These findings may reflect the influence of several factors, including suboptimal adherence, variability in patient characteristics, and the relatively high threshold set for clinical significance. A 2024 systematic review and meta-analysis of seven randomized controlled trials (*n* = 470) provided additional support for the role of exercise training in HFpEF. Exercise training was associated with significant improvements in 6 min walk distance and VO_2_ peak, although it had limited impact on central cardiac function parameters such as E/A ratio, E/e′, and left ventricular ejection fraction (LVEF). The most notable improvements were observed in the physical domains of HRQoL [64]. In response to this growing body of evidence, the American Heart Association and American College of Cardiology issued a scientific statement in 2023 endorsing supervised exercise training as a safe and effective therapy for patients with HFpEF. The statement emphasized that benefits are primarily mediated through peripheral adaptations, such as enhanced skeletal muscle metabolism and vascular function, rather than through changes in cardiac structure. It also highlighted the importance of individualized exercise prescriptions and strategies to support long-term adherence [65]. Looking ahead, ongoing trials such as the REHAB-HFpEF study are evaluating whether tailored physical rehabilitation can reduce hospital readmissions and mortality among patients recently hospitalized with HFpEF. As evidence continues to accumulate, expanding CR access to patients with HFpEF could help close a critical gap in heart failure care.

### 5.2. Older Adults

Frail and elderly patients represent another growing demographic in the heart failure population and have traditionally been considered too high-risk for participation in CR. However, a growing body of evidence challenges this perception, demonstrating that CR is not only feasible but also beneficial in improving clinical outcomes, physical function, and quality of life in this vulnerable group. The landmark REHAB-HF trial, a prospective multicenter randomized controlled study, enrolled patients aged ≥60 years who had been recently hospitalized with acute decompensated heart failure. Participants randomized to a tailored rehabilitation intervention showed significantly greater improvements in the Short Physical Performance Battery at 3 months compared to usual care (8.3 vs. 6.9; *p* < 0.001). The intervention, which included balance, strength, mobility, and endurance training, also enhanced independence and was safe across frailty strata [43]. Complementing these findings, the ongoing MACRO trial (Modified Application of CR for Older Adults) is evaluating the effects of personalized, flexible CR delivery on physical performance and patient-centered outcomes in older adults [66]. Such approaches are crucial, as conventional center-based CR may not be accessible or tailored to the needs of frail individuals. The concern that frailty might diminish CR benefits was specifically addressed in a large retrospective cohort study by Kamiya et al. [45], which included 3277 heart failure patients from 15 hospitals in Japan. CR was associated with significantly lower all-cause mortality and HF hospitalization (HR 0.77, 95% CI: 0.65–0.92; *p* = 0.003), including reductions in individual outcomes such as mortality (HR 0.67) and hospitalizations (HR 0.82). Stratified analysis by Frailty Index (FI) demonstrated that the benefits of CR persisted in patients with no frailty (FI < 0.21), mild (FI 0.21–0.31), and moderate frailty (FI 0.32–0.41), but not in those with severe frailty (FI ≥ 0.42), suggesting that while CR is broadly effective, its impact may diminish in the most debilitated patients, highlighting the need for further tailored approaches [45]. In addition to improving functional status, CR may also have a positive impact on quality of life and mental health in older adults. In one cohort study, elderly patients (≥65 years) completing CR showed significant improvements in exercise capacity (5.4 to 7.7 METs; *p* < 0.0001), body fat percentage (25.4% to 23.7%; *p* < 0.0001), and BMI (25.8 to 25.6 kg/m^2^; *p* < 0.01). Moreover, patients reported significant improvements in multiple domains of psychological health, including anxiety, depression, somatization, energy levels, mental health, and overall well-being (all *p* < 0.01) [67]. Collectively, this evidence dispels long-standing hesitations around referring frail or elderly patients to CR. With appropriately tailored programs, CR is not only safe but instrumental in restoring function, reducing hospitalizations, and enhancing quality of life for a population at high risk of decline.

### 5.3. Advanced HF

While patients with NYHA Class IV symptoms or Stage D heart failure have historically been excluded from large exercise trials due to safety concerns, emerging studies suggest potential benefits in carefully selected individuals. The REHAB-HF trial enrolled older, frail patients hospitalized with acute decompensated heart failure, some with Class IV symptoms, and showed that a tailored, multi-domain rehabilitation program significantly improved physical function and independence [42]. Similarly, the Rehab-VAD trial demonstrated that moderate-intensity aerobic training in patients with continuous-flow LVADs was safe and improved treadmill time, leg strength, and quality of life, despite minimal changes in VO_2_ peak [68]. More recently, the Ex-VAD trial, a multicenter randomized controlled trial in patients on long-term LVAD support (≥3 months), found that supervised exercise training did not significantly improve peak VO_2_ (*p* = 0.21), but resulted in meaningful gains in submaximal exercise capacity (mean 6MWT increase of 43 m, *p* = 0.03) and physical quality of life (KCCQ physical domain increase of 14.3 points, *p* = 0.007), with a favorable safety profile [69]. Observational data from a Medicare population further support these findings, showing that participation in cardiac rehabilitation after LVAD implantation was associated with a 23% reduction in rehospitalizations and a 47% reduction in one-year mortality (adjusted HR for mortality 0.53; 95% CI: 0.37–0.75; *p* < 0.001) [70].These findings support the feasibility of individualized rehabilitation strategies in advanced heart failure when medically appropriate.

### 5.4. Catheter-Based Valve Interventions (Transcatheter Aortic Valve Replacement, Transcatheter Mitral Valve Repair)

Transcatheter aortic valve replacement (TAVR) and Transcatheter Mitral Valve Repair (TMVR) are rapidly expanding treatment options for valvular heart disease, most commonly performed in older, frail adults who are often ineligible for surgery. While these minimally invasive procedures alleviate valve-related hemodynamic burden, many patients remain functionally impaired due to pre-procedural deconditioning, sarcopenia, and a high burden of comorbidities [71].

A growing body of evidence supports the role of CR in enhancing recovery after TAVR, demonstrating improvements in functional capacity, independence, and mortality. Among 62,628 Medicare beneficiaries, CR participation was associated with a 26% relative reduction in two-year all-cause mortality (HR 0.74, 95% CI 0.63–0.87, *p* < 0.01) [72]. In a systematic review and meta-analysis, Ribeiro et al. pooled data from five studies including 292 TAVR and 570 surgical aortic valve replacement (SAVR) patients. Functional gains were comparable between the two groups, with no significant differences in 6 min walk distance or Barthel Index scores, suggesting that both populations benefit equally from CR [73]. Similarly, Tarro Genta et al. (2017) [74] conducted a prospective study comparing CR outcomes in post-TAVR and post-SAVR patients. Despite TAVR patients having greater disability and fall risk at baseline, both groups experienced comparable improvements in exercise capacity and functional status, with TAVR patients even more likely to be discharged home after rehabilitation [74]. A more recent prospective study following 105 post-TAVR patients for 12 months found that those who completed inpatient CR had sustained improvements in 6 min walk distance and handgrip strength, along with notable gains in quality of life. Importantly, no device-related complications were observed, underscoring the safety and durability of these benefits even in older, high-risk patients [75]. Although less extensively studied, CR after TMVR has shown similarly encouraging results. In one of the few prospective studies available, inpatient CR was started approximately two weeks following TMVR placement in a group of older, frail adults. Participants experienced improvements in mobility, balance, and reduced reliance on assistive devices. No adverse device events were reported. The structured inpatient setting also allowed for early detection of clinical decline, adjustment of medical therapy, and attention to psychosocial needs. Several patients were able to secure home support services and disability benefits, emphasizing the broader functional and social value of CR in this setting [76].

Despite its clear benefits, CR (CR) remains significantly underutilized following TAVR. In one study, only 30.6% of eligible patients-initiated CR within 90 days of discharge. Participation rates varied widely across hospitals from 5% to 60% with much of this variation unexplained by patient characteristics (Sukul et al., 2023 [77]). Factors linked to lower participation include older age, Medicaid coverage, dialysis dependence, atrial fibrillation, limited mobility, transportation challenges, and reliance on caregivers [77]. Conversely, hospitals with higher patient satisfaction and a stronger emphasis on CR tend to have better enrollment rates [75]. In response to these barriers, alternative delivery models have been developed to improve accessibility. While inpatient CR remains standard, home-based and hybrid models are being explored. One pilot program at the Veterans Affairs Medical Center implemented a 12-week, physician-supervised home-based CR model for patients after TAVR. Although the study included a small number of participants, the program showed improvements in exercise capacity and physical functioning, supporting the feasibility and effectiveness of home-based CR for patients with limited mobility or geographic access [78]. As the evidence continues to expand, future efforts should prioritize improving referral processes, implementing flexible delivery options, and addressing systemic barriers that disproportionately affect older and medically complex patients. Multidisciplinary and accessible CR programs represent a key opportunity to support post-procedural recovery, improve quality of life, and reduce long-term morbidity and mortality in this growing population.

### 5.5. Pulmonary Hypertension (PH)

The role of exercise-based rehabilitation in PH was first established in 2006, when a randomized controlled trial demonstrated significant improvements in 6MWD, along with enhancements in functional class and peak oxygen uptake, in patients with severe chronic PH [79]. Since then, these findings have been confirmed in multiple studies and extended to include a broad range of PH populations, including those with congenital heart disease, chronic thromboembolic PH (CTEPH), and various other WHO group subtypes [80,81,82]. Most recently, a large multicenter randomized trial conducted across 10 European countries enrolled patients with pulmonary arterial hypertension (PAH) and CTEPH and reported improvements in 6MWD, as well as gains in WHO functional class, peak VO_2_, and mental health scores, without an increase in adverse events [83]. A 2023 Cochrane review synthesizing data from 14 randomized trials further reinforced these outcomes, showing a mean increase in 6MWD of 48.5 m, an average improvement in peak oxygen uptake of 2.07 mL/kg/min and significant improvements in both the SF-36 Physical Component Score (mean difference 3.98, 95% CI 1.89 to 6.07; *p* < 0.001) and Mental Component Score (mean difference 3.60, 95% CI 1.21 to 5.98; *p* = 0.004) [84]. Importantly, these programs were not associated with an increased risk of serious adverse events.

In recognition of this growing body of evidence, major professional societies including the European Society of Cardiology and European Respiratory Society and the American College of Chest Physicians have endorsed supervised exercise training as an important component of comprehensive care for selected patients with PH [85,86,87]. These guidelines emphasize the need for clinical stability, optimized medical therapy, and expert monitoring throughout rehabilitation. Both inpatient and outpatient programs have demonstrated benefit, although inpatient models may offer enhanced safety for higher-risk individuals [83,88]. Taken together, these findings reinforce that exercise-based rehabilitation is not only feasible and safe in appropriately selected patients with PH but also represents a valuable tool to improve functional status, quality of life, and overall risk stratification in a population with limited non-pharmacologic treatment options.

### 5.6. Cardio-Oncology and Cancer Survivors

CVD has become a leading cause of morbidity and mortality among cancer survivors [89]. This elevated risk arises from both the direct cardiotoxic effects of cancer therapies and indirect contributors including sedentary behavior, weight gain, and accelerated biological aging [90,91,92]. Survivors living five or more years beyond diagnosis have up to a 3.6-fold increased risk of cardiovascular mortality and significantly higher rates of hypertension, diabetes, and dyslipidemia compared to the general population [93]. Cardio-Oncology Rehabilitation (CORE), modeled after traditional cardiac rehabilitation CR, has been proposed to address this growing burden [94]. CORE integrates supervised exercise, cardiovascular risk factor management, nutrition counseling, psychosocial support, and education, all tailored to the unique needs of patients before, during, or after cancer therapy [94]. The 2021 AHA Scientific Statement endorsed CORE for high-risk individuals, including those with prior exposure to high-dose anthracyclines or chest radiation, a history of myocardial infarction or low ejection fraction, or the presence of multiple cardiovascular risk factors [95].

Emerging evidence supports this concept: observational studies have linked higher physical activity levels to reduced cancer-related and all-cause mortality, as well as fewer cardiovascular events among breast cancer survivors [96,97]. Randomized trials have further refined the evidence base. The TITAN trial, which evaluated breast cancer patients receiving anthracyclines, found no significant differences in left ventricular ejection fraction or cardiorespiratory fitness between CR and usual care [98]. However, low cardiotoxicity rates and suboptimal adherence may have limited the ability to detect benefits. In contrast, the more recent ENCORE trial randomized trial in early-stage breast cancer patients demonstrated that participation in a CORE program attenuated LVEF decline associated with anthracycline or HER2-targeted therapy and led to meaningful reductions in body mass index, particularly among obese individuals. The intervention was safe, well-tolerated, and associated with preserved physical performance and increased physical activity levels [99]. Specifically designed to evaluate the effects of a structured, multimodal rehabilitation program, the CORE trial compared a center-based cardiac rehabilitation program to a community-based exercise training model in cancer survivors at elevated cardiovascular risk [100]. The center-based program resulted in significantly greater improvements in peak VO_2_, blood pressure, BMI, quality of life, and physical activity levels. Adherence was also notably higher (90% vs. 68%), and greater improvements in cardiorespiratory fitness were associated with lower mortality, reinforcing the value of CR in survivorship care [100]. The benefits of CR may extend beyond cardiovascular outcomes. In the ERASE trial, men with prostate cancer under active surveillance who completed a HIIT program demonstrated improvements in functional capacity and a slower rise in prostate-specific antigen, suggesting a potential anti-proliferative effect of exercise [101]. These outcomes have also been supported in broader reviews. A systematic review of 25 randomized controlled trials involving hematopoietic stem cell or bone marrow transplant recipients found that structured exercise programs improved aerobic capacity, muscle strength, emotional well-being, and quality of life [102]. Similarly, a meta-analysis by Fakhraei et al. showed improvements in cardiorespiratory fitness and fatigue with CR-based interventions in cancer survivors, though many included studies were limited by risk of bias and inconsistent reporting [103].

While additional randomized trials are needed to refine protocols and assess long-term survival benefits, current evidence supports CORE as a safe, feasible, and promising strategy to reduce cardiovascular risk and enhance survivorship outcomes in this growing and vulnerable population.

### 5.7. Arrhythmias and Cardiac Implantable Electronic Devices (CIEDs)

CR is increasingly recognized as a beneficial intervention for patients with arrhythmias and CIEDs, including implantable cardioverter-defibrillators (ICDs) and cardiac resynchronization therapy (CRT) devices.

After ICD implantation, many patients reduce physical activity due to fear of shocks or uncertainty about exertion thresholds [104,105]. However, accumulating evidence supports CR as both safe and effective in appropriately selected patients. The HF-ACTION trial included 1053 patients with ICDs [106]. In a prespecified subgroup analysis, exercise training was not associated with increased ICD shocks, affirming CR’s safety in this population [106]. Subsequent smaller RCTs have shown that structured exercise improves cardiorespiratory fitness and is safe with a recent meta-analysis reporting a mean increase in peak VO_2_ of 1.98 mL/kg/min and a 47% reduction in the odds of ICD shocks (OR 0.53; 95% CI: 0.33–0.84; *p* = 0.007) with exercise training [107]. Large-scale observational data reinforce these findings. A retrospective study of 41,882 Medicare beneficiaries with ICDs found that higher device-measured physical activity was associated with a 44% lower risk of all-cause mortality, with the greatest benefit seen in CR participants [108]. Based on this evidence, both European and U.S. guidelines affirm that CR is safe for patients with ICDs when preceded by appropriate evaluation and risk stratification [109,110]. In CRT recipients, similar benefits have been observed. One of the largest trials, by Patwala et al., found that moderate continuous training initiated three months post-CRT improved both cardiac and skeletal muscle function [111]. Martens et al. also reported that participation in CR was associated with improved NYHA class, reverse remodeling, and reductions in mortality and heart failure hospitalizations [112]. Notably, gains in functional capacity have been observed even among patients with minimal improvement in LVEF [113]. A meta-analysis by Guo et al., which included seven RCTs in CRT patients, found that non-high-intensity training significantly improved peak VO_2_ (mean difference 3.05 mL/kg/min; 95% CI: 1.82–4.27; *p* < 0.001), LVEF (MD 4.97%; 95% CI: 3.23–6.71; *p* < 0.001), and quality of life [114]. High-intensity programs showed less consistent outcomes, potentially due to lower adherence [114]. These findings support moderate-intensity, individualized CR programming. Based on this evidence, both U.S. and European guidelines endorse CR for patients with CRT devices, while acknowledging the need for more device-specific trials [115,116].

Atrial fibrillation (AF), the most common sustained arrhythmia, represents another population where CR is gaining momentum. Early randomized controlled trials, including the OPPORTUNITY trial and the study by Malmo et al., demonstrated that HIIT significantly reduced AF burden and symptom frequency compared to moderate-intensity continuous training [117,118]. These studies also reported improvements in cardiorespiratory fitness and quality of life among patients with persistent and permanent AF [118]. A 2024 Cochrane review of 20 RCTs (*n* = 2039) further confirmed that exercise-based CR improves exercise capacity, reduces AF recurrence by 24% (relative risk 0.76; 95% CI: 0.62–0.94; *p* = 0.01), and alleviates AF-related symptoms [119]. However, its effect on all-cause mortality and adverse events remained uncertain due to limited follow-up and event reporting [119]. Real-world data offer additional insight: in a retrospective cohort of 23,894 patients with newly diagnosed AF, CR participation was associated with a 68% reduction in all-cause mortality (HR 0.32; 95% CI: 0.30–0.35; *p* < 0.001) and a 44% reduction in rehospitalization (HR 0.56; 95% CI: 0.53–0.59; *p* < 0.001) over 18 months [120]. Benefits were consistent across age, sex, and AF subtype [120]. Additional large epidemiologic studies have linked modest improvements in cardiorespiratory fitness or physical activity to lower risk of AF onset, progression, and cardiovascular death [121,122]. Both the 2024 European Society of Cardiology Guidelines and contemporary U.S. guidelines support lifestyle and exercise-based care in AF management, though formal CR referral is not yet standard [123,124].

## 6. Barriers to Access and Utilization

### 6.1. System-Level and Referral Barriers

Despite strong guideline recommendations and decades of demonstrated benefit, CR remains significantly underutilized worldwide. Global participation rates range from approximately 10% to 30%, with even lower rates among underserved populations and in low-resource settings [125,126]. The most consistently identified barriers include low referral rates and limited access which together represent a critical bottleneck in the CR care continuum [127].

Referral patterns are strongly influenced by hospital systems and infrastructure. Programs relying on manual referrals typically have lower enrollment compared to those with automatic, embedded processes [128,129]. A national cohort study in the U.S. revealed striking variation in CR enrollment by institution: patients discharged from high-performing hospitals were nearly twice as likely to attend CR compared to those from low-performing sites [130]. While approximately 30 percent of hospitals exceed the national average CR participation rate of 24 percent, fewer than 1 percent reach the recommended target of 70 percent participation across all eligible conditions [130]. Notably, 92 percent of CR-eligible patients and 96 percent of program participants are treated at hospitals that offer cardiac surgery, highlighting an important opportunity for strategic improvement within these institutions [130]. At the provider level, physician referral remains one of the strongest predictors of participation [131]. Yet, many clinicians remain unaware of CR’s clinical and economic benefits [132]. Time constraints, competing responsibilities, and unfamiliarity with updated guidelines contribute to under-referral [133]. To close these gaps, newer models using multidisciplinary strategies such as nurse-led, navigator-supported, or automated referral systems have demonstrated significant improvements in both referral and enrollment. One hospital implementing an opt-out referral system for post-PCI patients saw referrals increase by over 2000% [134]. A two-hospital system using EMR standardization and provider education increased referral rates from 51.2% to 87.1% in one year, while also eliminating racial disparities in referrals [135]. These findings underscore the potential of structured, system-level interventions to promote both utilization and equity.

Beyond referral, physical access remains a major barrier. Geographic disparities in program distribution and capacity particularly affect low- and middle-income countries (LMICs), which account for over 80% of global cardiovascular mortality [136]. A global survey found CR programs exist in only 54.7% of countries, with stark regional disparities: 80.7% of European countries offer CR compared to just 17.0% in Africa [127]. Moreover, even within regions, programs are often concentrated in urban tertiary centers, leaving rural and outlying populations underserved [127]. In the U.S., for example, many regions, particularly in the Southeast and rural areas, lack adequate CR infrastructure. Long wait times and travel distances over 24 km are associated with a 71% lower likelihood of participation [137]. Similar challenges have been reported internationally. A study from the Czech Republic found that distance was the most significant barrier to CR participation, with programs available in only three major cities and average one-way travel times of 50 min, well beyond the recommended threshold [138]. In LMICs, rural residents report similar obstacles, with distance and transportation difficulties frequently cited as primary barriers [136]. Importantly, emerging evidence highlights that program density, defined as the ratio of available cardiac rehabilitation centers to eligible patients, may better predict access inequities than travel distance alone [139]. In the U.S., regions like the West North Central and Mountain divisions show the highest CR initiation rates, while urban areas with limited centers and high patient volumes lag behind [139]. These underserved regions also house a disproportionate number of older adults, including Hispanic individuals aged ≥65, whose regional representation is nearly triple the national average [139]. While this is already concerning, it is worth noting that the U.S. is among the most densely covered countries, with an average of one CR spot per four patients, compared to one per 283 in Southeast Asia and one per 529 in Africa [127]. These stark global disparities further highlight the inequities in cardiac rehabilitation access and underscore the urgent need for strategic infrastructure investment and policy reform to close persistent geographic and demographic gaps.

Even when referral and access are addressed, financial barriers persist. CR programs face sustainability challenges globally. In the U.S., reimbursement varies by care setting and often fails to cover operating costs, particularly in rural and safety-net hospitals. A multicenter study found that CR programs lost an average of $429 per Medicare patient, with per-session costs exceeding reimbursement two- to threefold [140]. The COVID-19 pandemic further exacerbated this, shifting average margins from a $62 surplus to a $421 deficit per participant [141]. Internationally, the picture is similarly strained. In Europe, 26 countries report that 75 to 100 percent of their CR programs receive public funding, while in 10 countries, only 0 to 25 percent of programs are publicly supported. This highlights the uneven landscape of CR financing across the region [142]. For example, fewer than half of CR programs in Eastern Europe and Portugal receive government funding [142]. Moreover, most LMICs report that patients directly bear most of CR costs [136,143,144,145,146]. Although median per-patient costs are lower than in high-income countries, they remain unaffordable for many [136,143,144,145,146]. Public or insurance reimbursement, when available, is inconsistent and often inadequate [136,143,144,145,146]. Strengthening CR financing is essential to reduce disparities and improve outcomes as cardiovascular disease continues to rise globally.

### 6.2. Disparities by Age, Gender, Race/Ethnicity, and Socioeconomic Factors

Barriers to CR access are also deeply rooted at the patient level, with disparities observed across age, sex, race and ethnicity, as well as socioeconomic status. These inequities reflect broader structural challenges within the healthcare system and contribute to the unequal delivery of a highly effective secondary prevention intervention.

Sex and gender disparities in CR access and participation have been consistently documented. Women are 20% to 30% less likely to be referred to or participate in CR compared to men, even after adjusting for age and clinical factors [147]. Data from the APPROACH registry further reveal that, even when referred, women are less likely than men to attend or complete CR programs [148]. Contributing factors include higher rates of transportation challenges, caregiving responsibilities, and a lower perceived benefit from exercise-based interventions [149]. Beyond structural barriers, cultural norms and gender roles further limit women’s participation. In many countries, women face compounded obstacles such as modesty concerns, lack of women-only facilities, financial constraints, limited transportation, and household obligations that are less frequently reported by men [150,151]. These issues are often amplified by lower employment and education levels, which may hinder access to health information and supportive services [150,151]. Nevertheless, adaptive strategies are emerging. In the Arab world, for example, women-only classes are more frequently offered, and there is growing interest in home-based and telerehabilitation models to address cultural and logistical barriers [152,153]. Despite these innovations, the evidence base for women-tailored CR programs remains limited, underscoring the need for more inclusive design and targeted research.

Racial and ethnic disparities are similarly troubling. Black, Hispanic, and Asian patients are significantly less likely to be referred to or enroll in CR compared to White patients. In one Medicare analysis, minority patients were 20 to 40 percent less likely to initiate CR [154]. These disparities persisted even after controlling for insurance type, hospital characteristics, and clinical status, suggesting that other contributing factors such as language barriers, implicit bias, and a lack of culturally tailored care may drive unequal outcomes [155]. However, studies also demonstrate that once enrolled, Black patients complete CR at rates comparable to White patients [156]. Highlighting that improving referral and enrollment processes may be a particularly high-yield strategy in addressing racial disparities in CR participation. Cultural beliefs and religious values can also influence CR engagement [157]. For example, societal norms and family expectations may hinder the lifestyle changes needed after cardiac events [158]. In some communities, religious practices such as daily prayer or fasting have also been reported to conflict with CR schedules, leading to missed sessions [158,159,160]. These findings underscore the importance of culturally sensitive programming and flexible scheduling to accommodate diverse patient needs.

Age-related barriers further complicate equitable CR delivery. Participation rates decline steadily with advancing age, even though evidence suggests that older adults derive substantial benefits, particularly in maintaining independence, physical function, and quality of life [161]. Commonly cited obstacles include mobility limitations, cognitive decline, sensory impairments, lack of transportation, and insufficient caregiver support [161]. These factors contribute not only to lower rates of referral and enrollment but may also reflect provider biases and outdated concerns about safety or potential benefit in older adults [162,163]. Yet, when older adults are enrolled, they complete CR at rates similar to younger individuals and demonstrate significant improvements in physical function, quality of life, and even cognitive performance [162,164,165,166]. Home-based and hybrid CR models represent promising solutions for those who are unable to access traditional facility-based programs.

Lastly, socioeconomic factors exert a powerful and multifaceted influence on CR access and participation. Income level, education, and insurance status are strong predictors of both enrollment and completion [167,168,169]. In LMICs, cost remains one of the most significant barriers to both access and adherence [170]. Patients often pay up to 65% of CR costs out-of-pocket, compared to just 24% in high-income countries [143]. These expenses frequently exceed patients’ ability to pay. In Latin America, for example, the median out-of-pocket cost for a full course of CR ranges from PPP$146 in Venezuela to PPP$1095 in Brazil, with most programs requiring patients to shoulder some or all of these expenses [144,146]. Given that the average monthly income in many Latin American countries is below PPP$1000, CR often represents an entire month’s income, creating a substantial barrier to participation and adherence [171]. In the United States, similar financial challenges persist. A cohort study found that individuals with household incomes over $25,000 had 64% higher odds of participating in CR than those earning less than $15,000 (OR 1.68, 95% CI 1.17–2.42) [172]. Uninsured patients were 60% less likely to initiate CR within the first month (OR 0.39, 95% CI 0.21–0.71), and dual-eligible Medicare/Medicaid beneficiaries were significantly less likely to enroll than those with Medicare alone (OR 0.65, 95% CI 0.59–0.71) [4]. Cost-sharing requirements further exacerbate disparities: a full 36-session program may cost $1900–$2500 out-of-pocket [173]. A Michigan study showed that each $10 increase in copayments was associated with 0.41 fewer sessions attended, and the mere presence of a copay, regardless of amount, was linked to lower adherence [174].

Importantly, neighborhood-level deprivation also independently predicts lower CR utilization. In the Southern Community Cohort Study, only 8% of eligible patients enrolled, and those living in the most socioeconomically deprived areas were 58% less likely to participate than those in the least deprived areas (OR 0.42, 95% CI 0.27–0.66), even after adjusting for individual income and education [172]. This pattern is echoed internationally. For example, a Canadian study found that patients residing in low-income neighborhoods were significantly less likely to attend or complete CR compared to those in high-income neighborhoods [175]. Across global and local settings, financial and structural barriers disproportionately impact low-income individuals. These obstacles may prevent participation even when CR is medically indicated and technically covered by insurance. Tackling these systemic inequities is critical to expanding access and realizing the full preventive potential of cardiac rehabilitation.

## 7. Innovative Delivery Models and Future Directions

### 7.1. Alternative Delivery Models: Virtual, Home-Based, and Hybrid Programs

The emergence of virtual, home-based, and hybrid models of CR offers a transformative opportunity to overcome long-standing barriers in access and delivery. These innovative approaches, which incorporate telehealth platforms, remote monitoring, and flexible scheduling, have shown promising clinical outcomes while potentially reducing infrastructure costs and increasing scalability.

Early evidence suggests that alternative CR delivery models can achieve outcomes comparable to traditional center-based programs [139,176]. A 2022 Kaiser Permanente cohort study found that patients enrolled in home-based CR had 21% lower odds of hospitalization at 12 months compared to their center-based counterparts, with benefits observed across both low- and high-risk groups [177]. Similarly, a 2023 Cochrane systematic review concluded that home-based CR is non-inferior to center-based CR, showing no significant difference in total mortality (RR = 1.19, 95% CI: 0.65–2.16) or exercise capacity (SMD = −0.10, 95% CI: −0.24 to 0.04) over a 12-month period. Importantly, the cost per patient were similar between the two models [178]. Supporting these findings, an observational study from the Veterans Affairs system reported a 36% lower hazard of mortality among home-based CR participants during a median follow-up of 4.2 years [179]. Evidence is also emerging for hybrid CR models. A 2019 meta-analysis evaluated nine randomized controlled trials comparing hybrid and center-based CR and found significantly greater improvements in peak VO_2_ favoring the hybrid approach (mean difference +9.72 mL/kg/min) [180]. More recently, the 2025 Synchronous Tele-Hybrid Trial a randomized, noninferiority study demonstrated that a hybrid model combining in-person and tele-rehabilitation sessions produced comparable improvements in functional capacity, strength, and cardiopulmonary performance when compared to traditional CR, supporting its effectiveness across delivery settings [181]. In parallel, a multicenter real-world study of real-time, supervised cardiac telerehabilitation demonstrated high feasibility and patient satisfaction, with improvements in aerobic capacity, muscle strength, and reductions in kinesiophobia, along with nearly 70% adherence and few adverse event [182].

The COVID-19 pandemic accelerated the adoption of these models as widespread closures of in-person CR centers drove a need for remote alternatives. Virtual and hybrid programs became a critical lifeline, allowing patients to continue rehabilitation while adhering to public health guidelines. Notably, these approaches may help close persistent disparities in CR participation. Groups traditionally underrepresented in center-based CR, including older adults, women, racial and ethnic minorities, rural residents, and patients facing transportation or caregiving constraints, may find remote and hybrid models more accessible, potentially leading to higher enrollment [178,183,184].

A multicenter randomized trial in Chile, for example, found that a hybrid CR model was non-inferior to center-based CR in preventing cardiovascular events over 12 months. Participants, many of whom faced access challenges, experienced higher adherence with the hybrid approach, particularly in low-resource settings [185]. Similarly, a study comparing outcomes in younger and older adults participating in a 12-week hybrid CR program found comparable gains in grip strength, leg strength, and 6 min walk distance, affirming the model’s effectiveness across age groups [186]. In another longitudinal cohort of 753 medically complex women within the Kaiser Permanente Southern California system, participants in a home-based CR program had similar 12-month all-cause and cardiovascular hospitalization rates as those in center-based programs (17% vs. 18%) [184]. Importantly, these outcomes were achieved despite the home-based group having greater comorbidities and longer travel distances, highlighting the model’s potential to bridge geographic and clinical access gaps.

Together, these studies illustrate the clinical viability and equity-enhancing potential of virtual and hybrid CR. Yet, to date, only 9% of CR programs worldwide, across 24 countries, offer some form of hybrid model [136]. Broader implementation will require addressing several key challenges. Equitable access to technology is essential to avoid reinforcing existing disparities. Additional priorities include training healthcare teams in virtual care delivery, sustaining patient engagement in remote settings, and integrating program data into electronic health records. Standardized protocols and sustained reimbursement will also be critical to ensuring long-term viability and expansion.

### 7.2. Expanded Eligibility and Reimbursement

CR is a highly cost-effective intervention that reduces mortality, cardiovascular events, and hospital remissions, while improving quality of life and return to work [145]. Its clinical benefits are consistent across geographic regions, and in LMICs, where provider salaries and infrastructure costs are lower, CR may even be cost-saving [145]. Studies estimate that delivering CR in LMICs may cost half as much as in high-income countries while offering equivalent health outcomes [144,145]. However, despite its favorable cost-effectiveness profile, CR remains underfunded and underprioritized in many countries. As a result, even cost-effective care may remain inaccessible due to limited government investment, lack of reimbursement infrastructure, and competing healthcare priorities [144].

Encouragingly, some countries have begun to address the reimbursement gap in cardiac rehabilitation. In South Korea, the inclusion of CR under national health insurance in February 2017 led to a fourfold increase in program participation (odds ratio [OR] 3.99, 95% CI: 2.89–5.51). Following this policy change, out-of-pocket costs decreased to approximately $18 USD per exercise session and $9 USD per education session, resulting in a marked uptick in utilization [187]. Israel has proposed designating CR as a national health quality indicator and expanding referrals beyond classical indications [188]. Germany supports reimbursement for risk factor counseling, and the United Kingdom is exploring pay-for-performance models tied to measurable outcomes, such as improvements in exercise capacity [188]. A significant milestone in Egypt is the establishment of the country’s first public-sector CR program [189]. Additional efforts are underway across the region; for example, the Ministry of Health in Saudi Arabia is actively working to integrate CR services into the public healthcare system [190]. While most LMICs globally still lack established reimbursement pathways for hybrid CR, some high-income countries such as the United Kingdom have begun to support these programs by piloting and implementing them in selected regions [145,191]. These global efforts provide adaptable frameworks to improve both access and reimbursement.

In the U.S., Medicare policy plays a central role in determining access to and sustainability of CR programs. In 2016 alone, more than 366,000 Medicare beneficiaries were eligible for outpatient CR, highlighting the influence of federal coverage decisions in shaping national participation rates [4]. In recent years, incremental but meaningful policy shifts have broadened access for high-risk populations. A major milestone occurred in 2014, when Medicare expanded eligibility to include patients with HFrEF, significantly increasing the pool of qualified participants [192]. However, many patients, such as those with HFpEF and other high-risk cardiovascular conditions, remain ineligible for coverage, limiting access for large segments of the population who may benefit from CR. In addition to eligibility gaps, financial challenges remain a major constraint. Recognizing this, policymakers have begun experimenting with value-based reimbursement models that incentivize CR delivery and outcomes. In 2011, Medicare increased the per-session CR reimbursement from $68 to $104 (a nearly 180% increase), though this alone did not substantially raise enrollment [193]. During the COVID-19 Public Health Emergency, CMS launched the Hospital Without Walls initiative, which temporarily authorized reimbursement for virtual CR and allowed remote physician supervision. These flexibilities enabled the rapid expansion of hybrid and home-based delivery models and laid the groundwork for their continued adoption [194]. More recently, in 2025, CMS finalized a 2.9% update to the Hospital Outpatient Prospective Payment System. This market-basket-based increase applies to all outpatient services, including CR delivered in hospital-based programs, and represents a modest but meaningful step toward sustaining program infrastructure [195].

Moving forward, sustainable reimbursement will depend on expanding eligibility criteria, permanently recognizing alternative delivery models, and ensuring adequate payment structures. Without targeted reform, the full potential of CR will remain unrealized. Importantly, there is also a need for clinical trials to evaluate the long-term economic sustainability of CR, including its potential to generate health system savings and reduce cardiovascular recurrences.

### 7.3. Policy Reform and Quality Improvement Initiatives

A number of global and regional initiatives have laid the groundwork for CR within health systems worldwide, particularly in LMICs [136]. The World Health Organization’s Rehabilitation 2030 Initiative offers an overarching framework to integrate rehabilitation, including CR, into universal health coverage and primary care services, especially in underserved regions [136]. Complementing this, the International Council of Cardiovascular Prevention and Rehabilitation (ICCPR) launched a Global CR Registry to support quality improvement through standardized performance metrics and enable international benchmarking [126]. In parallel, ICCPR established a global CR Foundations Certification program, which has trained over 3000 professionals across 35 countries, with scholarships targeting LMIC practitioners and content translated into multiple languages to facilitate wider dissemination [196]. ICCPR has also catalyzed research and implementation projects in LMICs, including randomized hybrid CR trials, adaptation of UK home-based models [126,197,198,199]. Recent initiatives include close collaboration with leaders in Africa to support CR capacity-building and expand program reach [126]. In Europe, the CHRODIS+ Joint Action promotes integrated care and secondary prevention strategies across EU member states, reinforcing CR’s role in chronic disease management [200]. Nationally, countries such as Germany, Japan, and Canada have incorporated CR into reimbursement policies or health system performance indicators [136,201,202]. The United Kingdom’s Heart Manual Program and Canada’s nurse-led home-based models are examples of scalable, evidence-based approaches that have been widely adopted and reimbursed at the national level [203,204,205]. These initiatives reflect a growing global consensus on the importance of CR and the need to embed it within broader chronic care strategies. Several LMICs, including India, Iran, China, and nations across Latin America and sub-Saharan Africa, have begun adapting low-cost, community-based, or hybrid CR models to overcome resource constraints [126,136,185,206]. Many of these efforts have been supported by ICCPR and other international organizations, highlighting scalable strategies for expanding CR access globally [145].

High-income countries have also made targeted efforts to improve CR utilization. In the United States, the Million Hearts Initiative, co-led by the CDC and CMS, positioned CR as a key strategy in secondary prevention, with an ambitious goal to increase participation from 20% to 70% [207]. This catalyzed the formation of the Million Hearts Cardiac Rehabilitation Collaborative, a national network of nearly 400 individuals from over 200 organizations [207]. The group meets regularly to support cross-sector learning, coordinate quality improvement, and disseminate evidence-based tools [207]. Among its most notable contributions is the Cardiac Rehabilitation Change Package, which offers hospitals actionable strategies to improve participation, including automatic referral systems, culturally responsive care, and integration of hybrid models [207]. Federal investments have further accelerated progress. The NIH has dedicated over $5.5 million to research on home-based and hybrid CR models, particularly for underserved populations [208]. Public campaigns like “Live to the Beat” have raised awareness of CR’s benefits, while value-based insurance design models are reducing patient cost-sharing to improve access [209]. Federal policy has continued to evolve in support of flexible CR delivery. Building on the Hospital Without Walls initiative launched during the COVID-19 pandemic, which temporarily authorized facility-based, hybrid, and remote models, recent bipartisan legislation such as the Sustainable Cardiopulmonary Rehabilitation Services in the Home Act (S.3021) aims to make coverage for home-based and virtual CR permanent [210]. In parallel, CMS enacted a regulatory change effective 1 January 2024, that expands supervision eligibility. Whereas CR once required direct physician supervision, the new policy allows physician assistants, nurse practitioners, and clinical nurse specialists to supervise services, provided they remain immediately available [211]. By broadening the pool of eligible supervisors, this policy aims to improve program availability, particularly in rural and underserved areas where physician staffing may be limited. The Increasing Access to Quality Cardiac Rehabilitation Care Act (S.248/H.R.783) seeks to make this change permanent by making it into federal law [212]. In addition, the Sustaining Outpatient Services Act would allow hospitals to relocate or open new CR and pulmonary rehabilitation clinics in underserved areas without triggering site-neutral payment rules [207]. Currently, moving a program off-campus can result in significantly lower reimbursement. The bill would preserve hospital-level reimbursement rates even when services are delivered at new or relocated sites, making program expansion more financially feasible.

At the state level, Michigan has emerged as a national model. Through partnerships with the Blue Cross Blue Shield Cardiovascular Consortium, the Michigan Value Collaborative, and the Michigan Cardiac Rehab Network, the state collects standardized clinical data, disseminates best practices, and works to reduce unwarranted variation in care [213]. Notably, Michigan achieved a PCI-to-CR referral rate of 91% in 2023, translating to approximately 24,000 referrals that year [214]. These efforts, supported by educational toolkits and hospital benchmarking, earned the 2023 Eisenberg Patient Safety and Quality Award. This model highlights how coordinated regional strategies can drive large-scale quality improvement and may serve as a blueprint for national replication.

Together, these international and domestic initiatives demonstrate that expanding CR access is not only feasible but essential. As countries confront rising cardiovascular disease burdens, embedding CR into health systems through policy, workforce expansion, and innovative delivery models will be critical to advancing global cardiovascular health and equity.

## 8. Conclusions

CR is a cornerstone of cardiovascular secondary prevention, offering consistent and meaningful benefits across a growing spectrum of diseases, procedures, and patient populations. From improvements in functional capacity and quality of life to reductions in mortality and hospital readmissions, the evidence base is both broad and deep. Yet despite Class I guideline recommendations, CR remains severely underutilized, particularly among women, older adults, underserved populations, and those with limited access to traditional facility-based programs. As cardiovascular disease evolves into a chronic, heterogeneous condition affecting millions, CR must evolve with it. Expanding flexible delivery models alongside reimbursement reform, workforce expansion, and targeted policy initiatives can help bridge long-standing gaps in access. Ultimately, realizing the full potential of CR will require a shift from viewing it as a supplemental service to recognizing it as an essential, adaptable, and equity-driven component of modern cardiovascular care.

## Abbreviations

The following abbreviations are used in this manuscript:

AACVPR	American Association of Cardiovascular and Pulmonary Rehabilitation
ACC	American College of Cardiology
AHA	American Heart Association
CAD	Coronary Artery Disease
CMS	Centers for Medicare & Medicaid Services
CR	Cardiac Rehabilitation
ESC	European Society of Cardiology
HFpEF	Heart Failure with Preserved Ejection Fraction
HFrEF	Heart Failure with Reduced Ejection Fraction
MI	Myocardial Infarction
TAVR	Transcatheter Aortic Valve Replacement
TMVR	Transcatheter Mitral Valve Repair
VEGF	Vascular Endothelial Growth Factor
